# Supplementary material for: Association of red and processed meat consumption with cancer incidence and mortality: An umbrella review protocol
Source: PLoS One. 2025 Mar 13;20(3):e0315436. doi: 10.1371/journal.pone.0315436 (PMC11906087; doi:10.1371/journal.pone.0315436)
Supplement: Attachment — Submitted filename: Response to Reviewers.docx [file pone.0315436.s002.docx]

Umbrella reviews are systematic collections and assessments of multiple SRMAs done on a specific research topic. The two most common applications of umbrella reviews deal with treatment effects of interventions and epidemiological associations of exposures. Umbrella reviews have to follow several steps like definition of research question. Development of search algorithm, definition of inclusion and exclusion criteria, systematic literature review, data extraction, statistical analysis of eligible meta-analysis, grading strength of evidence, sensitivity analysis, upgrade or down grade of ranking, and discussion of potential bias and related issues. In the figure one few of these logical sequences were missed.

**Response:** Thank you very much for your comments. Figure 1 in the paper is a flow chart showing the literature search and selection, which is developed according to the official PRISMA website.

18: Why are authors mentioned to following 5 search engines and two registers. These should not be limited. Whatever is available, one can use.

**Response:**Thank you very much for your comments. The protocol of meta or umbrella analysis is an operational standard to improve the transparency and objectivity of meta-analysis. These 5 search engines and two registers were selected to define the operational process for our team to conduct meta-analysis in the future. According to AMSTAR-2, this is sufficient to identify the available literature.

19-20 Methods: We will independently, …………………..from inception to 2024

The time period mentioned here is from inception to July 2024. When the inception occurs and what the total time (months/years) needs to be mentioned in the protocol.

**Response:**Thank you very much for your comments. We have revised the article in detail according to your comments.

The time range here refers to the expected time range of literature retrieval. We expect the literature retrieval time to be from the establishment of the database to July 2024, while we add the statistical analysis time in the abstract: August 2024 to December 2024(page1, line 22-23). .

27-29: According to the ………………….highly suggestive, suggestive (repetitive word may be deleted) or weak evidence.

**Response:**Thank you very much for your comments. Highly suggestive and suggestive are standard terms based on evidence confidence evaluation criteria. Highly suggestive and suggestive represent two different levels.

Conclusions:

31-34: Conclusions need to be changed. The main aim of protocol publication is to get feed back/suggestions from the reviewers to develop a standard protocol before its publication and after publication, it should guide this protocol to take up similar research by any researcher(s) by following meticulously this standard protocol.

**Response:**Thank you very much for your comments. We have revised the article in detail according to your comments.

The conclusion of the article has been revised.

58-60: In addition ……………… in different studies. Please provide some references

**Response:**Thank you very much for your comments. We have revised the article in detail according to your comments.

We have added references 10-12 to the corresponding part of the article.

96-99: This sentence is not clear, needs to be rewritten.

Thank you very much for your comments. We have revised the article in detail according to your comments.

**Response:**We have changed the content of the article to: Systematic reviews that only included cohort studies and case-control studies and excluded RCTs and cross-sectional studies (CSSs) are eligible.

114: Meta analysis ………………. evidence synthesis. It needs to be mentioned what is the cut point taken as the sufficiency of the data?

**Response:**Thank you very much for your comments. We have revised the article in detail according to your comments.

We have changed the content of the article to: meta-analyses without available data for evidence synthesis.

125-126 Please give reference to this sentence Fig 2.

Fig 2.

What is the basis of RR defined as highest or lowest for various cancer incidents versus consumption of meat and processed meat?

**Response:**Thank you very much for your comments. We have revised the article in detail according to your comments.

We have given reference to this sentence Fig 2.

RR defined as highest or lowest for various cancer incidents is self-defined from the article in reference 8.

Discussion: Maybe be fine pruned further.

**Response:**Thank you very much for your comments. We have revised the article in detail according to your comments.

We have streamlined the discussion.

Journal Requirements:

2. We note that the grant information you provided in the ‘Funding Information’ and ‘Financial Disclosure’ sections do not match.

Please state what role the funders took in the study.

**Response:**Thank you for your comments. In the review comments, we add funders' help to the article, mainly including financial support for the use of search tools and the efficient operation of the team(page 18, line 244-256).

Comments to the Author

4.Have the authors described where all data underlying the findings will be made available when the study is complete?

**Response:**The data used in our article comes from public databases, i.e., PubMed, Embase, Web of Science, Scopus, and the Cochrane Library.
